# Needle tenoscopy of the digital flexor tendon sheath in a standing equine cadaver model using a novel approach and guided palmar/plantar annular ligament desmotomy

**DOI:** 10.1111/vsu.14213

**Published:** 2025-02-06

**Authors:** Louise J. Breen, John D. Stack, Alex M. Gillen, Chris M. Baldwin

**Affiliations:** ^1^ The Philip Leverhulme Equine Hospital, Institute of Infection, Veterinary and Ecological Science University of Liverpool Liverpool UK

## Abstract

**Objective:**

(1) To evaluate the feasibility of needle tenoscopy of the digital flexor tendon sheath (DFTS) using basisesamoid (BS) and proximolateral (PL) approaches in a standing equine cadaver model. (2) To report visualization of intrathecal DFTS anatomy via both approaches. (3) To determine the efficacy of needle scope‐guided palmar/plantar annular ligament (PAL) desmotomy. (4) To report any iatrogenic damage associated with the procedure.

**Study design:**

Ex vivo experimental.

**Sample population:**

Ten equine cadaver limbs.

**Methods:**

Limbs were placed in a Kimzey leg‐saver splint and needle tenoscopy was performed using the BS and PL approaches. Two European College of Veterinary Surgeons (ECVS) Diplomates assessed and categorized intrathecal site visualization as poor, partial, or excellent. Needle scope‐guided PAL desmotomy was performed after DFTS exploratory needle tenoscopy. Limbs were dissected and examined for the presence of iatrogenic damage and completeness of PAL desmotomy. A Wilcoxon signed‐rank test was used to compare visualization scores for both approaches.

**Results:**

Needle tenoscopy of the DFTS in a standing model was feasible from both BS and PL approaches. Excellent visualization of clinically significant intrathecal anatomy within the fetlock canal was achieved from both approaches (*p* ≤ .001), with minimal iatrogenic damage. The PL approach allowed more structures to be visualized than the BS approach (*p* = .025). All PAL desmotomies were completed without associated iatrogenic damage.

**Conclusion:**

Needle tenoscopy of the DFTS in a standing model provided excellent visualization of intrathecal sites within the fetlock canal. It facilitated complete PAL desmotomy.

**Clinical significance:**

Needle ten0oscopy can be used to assess the DFTS and to guide PAL desmotomy in a standing horse.

**Abbreviations**
BS: Basisesamoid
DDFT: Deep digital flexor tendon
DFTS: Digital flexor tendon sheath
IQR: Interquartile range
MF: Manica flexoria
MRI: Magnetic resonance imaging
PAL: Palmar/Plantar annular ligament
PL: Proximolateral
SDFT: Superficial digital flexor tendon sheath
Sub‐MF: Sub‐manica flexoria
VREC: Veterinary research ethics committee

## 1 INTRODUCTION

The digital flexor tendon sheath (DFTS) encases the superficial digital flexor tendon (SDFT), deep digital flexor tendon (DDFT), manica flexoria (MF), and associated structures, including manica, plicae, mesotenons, and vinculae.^1^ The SDFT and DDFT travel through the fetlock canal, passing between the palmar/plantar annular ligament (PAL), proximal sesamoid bones, intersesamoidean ligament, and palmar/plantar scutum.^1^ Common nonseptic pathologies of the DFTS include longitudinal tears of the SDFT and DDFT,^2^, ^3^, ^4^, ^5^, ^6^, ^7^ tears of the MF,^3^, ^4^, ^5^, ^8^, ^9^, ^10^ and PAL desmopathy.^3^, ^10^, ^11^ Multiple lesions within a DFTS are common, with two or more lesions identified in 75% of tenoscopic examinations.^3^ Nonsurgical diagnostic modalities of the DFTS include ultrasonography,^2^, ^4^, ^12^, ^13^ contrast ultrasonography,^14^ contrast radiography,^9^, ^10^ contrast CT,^15^ and magnetic resonance imaging (MRI).^16^ Despite the extensive array of imaging modalities available, tenoscopy is still considered the gold‐standard imaging modality for assessment of the DFTS intrathecal anatomy and allows concurrent surgical intervention.^3^

Diagnostic needle endoscopy in the standing equine patient has been used for the assessment of the metacarpo/metatarsophalangeal joints,^17^ femorotibial joints,^18^ antebrachiocarpal and middle carpal joints,^19^ cervical articular process joints,^20^ scapulohumeral joints,^21^, ^22^ bicipital bursa,^21^ carpal sheath,^23^ and navicular bursa.^24^ Needle endoscopy of the DFTS has been described in a cadaver study replicating lateral recumbency but poor visualization and a lack of rigidity led the authors to conclude that this was not a suitable diagnostic imaging modality.^25^ As far as the authors of the current study are aware, there are no reports in the literature describing the use of a nanoscope in tenoscopy or in equine surgery more broadly.

The aims of this study were (1) to evaluate the feasibility of needle tenoscopy of the DFTS in a standing equine cadaver model from a basisesamoid (BS) and a proximolateral (PL) approach; (2) to report visualization of intrathecal anatomy, via both approaches; (3) to determine the efficacy of needle scope‐guided PAL desmotomy; and (4) to report any iatrogenic damage from either the needle tenoscopy or PAL desmotomy. It was hypothesized that (1) needle tenoscopy of the DFTS in a cadaveric standing‐horse model would be feasible from both BS and PL approaches; (2) needle tenoscopy would allow visualization of the DFTS clinically relevant intrathecal anatomy from either a BS or PL approach; (3) needle tenoscopy would facilitate complete PAL desmotomy, and (4) needle tenoscopy and needle scope‐guided PAL desmotomy would result in minimal iatrogenic damage to intrathecal structures.

## 2 MATERIALS AND METHODS

### 2.1 Preoperative planning and surgical procedure

The University of Liverpool's veterinary research ethics committee approved the study (approval number VREC1199). Ten equine cadaver limbs (five forelimbs and five hindlimbs) were collected from an equine abattoir and were subjectively categorized as either Thoroughbred/Warmblood or Cob/Draught breeds. Reasons for euthanasia were unknown, so only limbs with no obvious abnormalities of the DFTS on visual inspection and palpation were included in the study. Limbs were transected at the distal radius or distal tibia before being frozen and stored at −20°C. Limbs were thawed over 12 h to room temperature prior to tenoscopy. Limbs were clipped with a number 40 clipper blade and prepared aseptically with a 2% chlorhexidine solution followed by isopropyl alcohol, from the coronary band to the proximal metacarpus/metatarsus.

To mimic conditions in which the procedure could be performed in the standing horse, the cadaver limbs were affixed within a standard‐size Kimzey “tiptoe” leg‐saver splint with the fetlock joint held in partial flexion (Kimzey Welding Works, Woodland, California). The limbs were taped in place at the level of the heel bulbs and proximal metacarpal/metatarsal region with general‐purpose adhesive tape (Tikkitape, Newbury, England).

**FIGURE 1:**
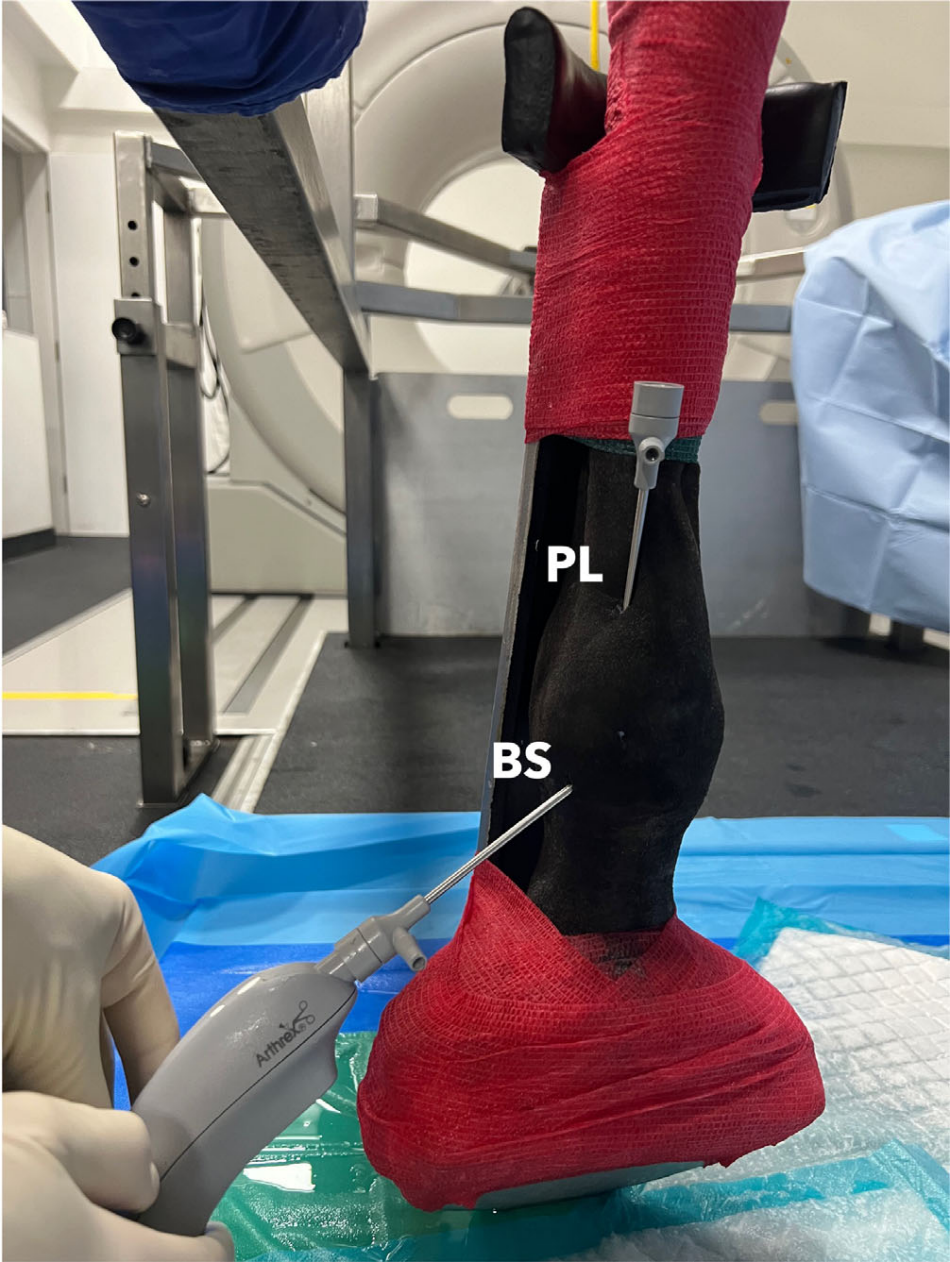
The plantarolateral aspect of the metatarsal region, indicating the position of the traditional basisesamoid and the novel proximolateral portal for tenoscopy of the digital flexor tendon sheath.

The DFTS was distended with 0.9% saline (Aquafarm, York, England), and an 18‐gauge 38 mm needle at the distal palmar/plantar pastern outpouching of the DFTS at the level of the second phalanx, until moderate distension of the lateral DFTS outpouching between the PAL and proximal digital annular ligament was observed, and the total volume required for distention was recorded. Two ECVS Diplomates who were experienced in traditional tenoscopy, performed all procedures and PAL desmotomies using needle tenoscopy (CMB and JDS). All portals were 3.5–4 mm long and created with a No. 11 scalpel blade. The BS approach portal was located between the PAL and the proximal digital annular ligament, 5 mm palmar/plantar to the palmar/plantar neurovascular bundle on the lateral aspect of the limb (Figure 1).^26^ The cannula was advanced proximally through the fetlock canal aiming for the proximodorsal recess of the DFTS. The PL approach portal was created in the same location as that described for ultrasound‐guided injection of the DFTS.^27^ The portal was located at the level of the proximal DFTS, 5 mm palmar/plantar to the neurovascular bundle. The cannula was advanced distad, attempting to situate the cannula dorsal to the DDFT and MF (Figure 1). The approach that was used first alternated between limbs. The BS approach was performed first in legs 1, 3, 5, 7, 9 followed by the PL approach. The PL approach was performed first in legs 2, 4, 6, 8, 10 followed by the BS approach. All limbs were operated on the same day.

A rigid, high flow, 10° angled, 3.4 mm outer diameter cannula and blunt conical obturator was used (Arthrex catalogue code: AR‐3210‐0051) and the number of attempts to enter the DFTS recorded. The obturator was replaced by a flexible, 0° working angle, 1.9 mm optic needle scope (NanoScope), with a 120° field of view and a 95 mm working length (Arthrex catalogue code: AR‐3210‐0040; NanoScope; Arthrex GmbH, Munich, Germany). Once the needle scope was situated within the DFTS fluid ingress was controlled using an Endomat arthroscopy pump (Karl Storz Endoscopy Ltd., Slough, England) and the minimum and maximum distension pressure was recorded.

### 2.2 Intraoperative visualization

The DFTS was divided into two regions: “fetlock canal” and “proximal DFTS.” The fetlock canal was defined as the region between the distal border of the PAL and the distal border of the MF. The proximal DFTS region was defined as the region from the distal border of the MF to the proximal reflection of the DFTS. The pastern region of the DFTS was not assessed because pathology is less commonly encountered in this region than in the fetlock canal.^2^, ^3^, ^4^ Tenoscopy was performed and visualization of the following sites was assessed and scored by two ECVS Diplomates (CMB and JDS) to reach a consensus score: dorsal, palmar, medial, and lateral borders of the SDFT and DDFT within the fetlock canal and proximal DFTS; proximal and distal border of the PAL; medial, lateral, and distal border of the MF; the mesotenons of the DDFT and the mesotenon of the SDFT (Table 1).

**TABLE 1:** Sum visualization scores for each of the 24 individual sites assessed during needle tenoscopy of the digital flexor tendon sheath (DFTS) from a basisesamoid (BS) and a proximolateral approach (PL). Scores were categorized as poor 0–6 (red), partial 7–13 (yellow), or excellent 14–20 (green). For the submanica flexoria (sub‐MF) approach (n = 3), sum visualization scores were classified as poor (0–2, red), partial (3–4, yellow), or excellent (5–6, green).

	Digital flexor tendon sheath																							
	Within fetlock canal								Proximal DFTS								Other structures assessed							
	Superficial digital flexor tendon				Deep digital flexor tendon				Superficial digital flexor tendon				Deep digital flexor tendon				Palmar annular ligament		Manica flexoria			Mesotenon		
	Dorsal	Palmar	Medial	Lateral	Dorsal	Palmar	Medial	Lateral	Dorsal	Palmar	Medial	Lateral	Dorsal	Palmar	Medial	Lateral	Distal border	Proximal border	Distal margin	Medial margin	Lateral margin	Medial DDFT	Lateral DDFT	SDFT
BS *n* = 10	20	19	13	20	20	20	6	19	8	0	0	6	12	7	2	5	19	0	20	8	11	0	0	19
PL *n* = 10	16	16	20	20	20	18	13	19	0	15	5	20	2	0	2	3	20	18	20	17	19	0	0	18
Sub‐MF *n* = 3	6	0	6	6	6	6	5	6	6	0	4	6	6	6	5	6	0	0	6	5	6	6	6	0

A visualization scoring system was utilized consisting of (0) no ability to visualize the structure or position the arthroscope in the desired location; (1) incomplete visualization, or (2) complete visualization of the structure.^28^ Both surgeons came to a consensus score for each structure during the procedure. The sum visualization scores generated a maximum 20‐point scoring system for each structure for all 10 limbs. Based on the sum score, the overall visualization for each structure was categorized as poor (sum score 0–6), partial (sum score 6–13), or excellent (sum score 13–20) (Table 1).

If during the PL approach the cannula was inadvertently placed through the proximal synovial sheath areolar reflection of the MF so that the needle scope was positioned between the DDFT and the MF, this was considered a third approach—submanica flexoria (sub‐MF). Visualization scores were recorded before the needle scope was withdrawn and positioned dorsal to the MF as for a standard PL approach.

The total time for portal creation and needle tenoscopy was recorded for BS, PL, and sub‐MF approaches.

### 2.3 Palmar/plantar annular ligament desmotomy

Following tenoscopy from both BS and PL approaches, the needle scope was positioned in the proximal DFTS via the PL portal. A 10–12 mm instrument portal was created with needle optimization, palmar to the neurovascular bundle, distal to the needle scope and proximal to the proximal border of the PAL. A curved hook knife (Reiter Leibinger, Muehlheim, Germany) was then inserted into the DFTS (Figure 3A) and under needle scopic visualization the hook knife was directed distad through the fetlock canal to the distal border of the PAL (Figure 3B). The hook knife was rotated to engage the distal border of the PAL before being drawn proximally transecting the PAL under needle scopic visualization (Figure 3C). The number of attempts to transect the PAL and total time to perform the PAL desmotomy were recorded in all limbs (Table 2).

**TABLE 2:** Findings associated with needle scope‐guided palmar/plantar annular ligament (PAL) desmotomy in all limbs (*n* = 10).

Limb	FL/HL	Attempts to make portal	Total time to create portal and perform PAL desmotomy	PAL thickness (mm)	Attempts to transect PAL	Complete PAL transection?
1	FL	1	5 min, 40 s	2	1	Yes
2	FL	2	9 min, 27 s	2	1	Yes
3	HL	1	3 min, 3 s	3	2	Yes
4	HL	1	4 min, 33 s	2	1	Yes
5	FL	1	9 min, 24 s	2	1	Yes
6	HL	1	9 min, 12 s	2	2	Yes
7	HL	1	6 min, 18 s	3	1	Yes
8	HL	1	6 min, 57 s	3	1	Yes
9	FL	1	7 min, 28 s	2	1	Yes
10	FL	1	7 min, 18 s	3	1	Yes

### 2.4 Iatrogenic damage, extravasation, and dissection

Extravasation at the portal sites was assessed at the end of the procedure and graded as none, mild, moderate, or severe.^28^ Following needle scope tenoscopy and PAL desmotomy, all limbs underwent gross anatomical dissection. The portals were assessed for proximity to the palmar/plantar digital neurovascular bundle and the portal location was recorded as correct or incorrect. The PAL desmotomy was recorded as complete or incomplete and PAL thickness was measured using electronic calipers and recorded (millimeters). All intrathecal structures were inspected for iatrogenic damage and graded for severity—none (no apparent iatrogenic damage), mild (<2 in number, <2 mm deep, <10 mm in length), moderate (2–4 in number, 2–3 mm deep, 10–20 mm in length), or severe (>5 in number, ≥4 mm deep, >30 mm in length).

### 2.5 Statistical analysis

Descriptive analysis was performed using Excel (Microsoft, Reading, England). Statistical analysis was performed with SPSS (IBM, Armonk, New York). Data were assessed for normality using Shapiro–Wilks tests. Visualization scores were compared between PL and BS approaches, and fetlock canal and proximal DFTS using Wilcoxon signed‐rank tests. Time taken to perform needle tenoscopy from a BS and a PL approach was also compared using a Wilcoxon signed‐rank test. Normally distributed continuous variables are presented as means with standard deviations and ranges, and those with non‐normal distribution as median with interquartile range (IQR). Statistical significance was set at *p* ≤ .05.

## 3 RESULTS

### 3.1 Preoperative planning and Surgical procedure

Of the 10 limbs, eight were Thoroughbred/Warmblood and two were Cob/Draught type. All DFTS were successfully distended on the first attempt with a mean volume of 52.5 min 1 s (SD ± 15.86; range 30–90). Needle tenoscopic entry into the DFTS was successful on the first attempt in all BS approaches and, 6/10 PL approaches. One PL approach required one additional attempt, and in three other limbs, the needle scope was placed sub‐MF before being withdrawn and positioned dorsal to the MF.

The mean time for needle tenoscopy from a BS approach was 8 min, 54 s (SD ± 3 min, 4 s; range 4 min, 38 s–14 min, 23 s); from a PL approach the mean was 6 m 46 s (SD ± 1 min, 51 s; range 4 min, 13 s–9 min, 39 s), and for sub‐MF approach 5 min, 48 s (SD ± 1 min, 30 s; range 4 min, 30 s–7 min, 55 s). There was no statistical difference in the time taken between the BS and PL approach (*p* = .86). Minimum arthroscopic fluid pressure was 50 mmHg for all approaches, and median maximal pressure was 50 mmHg for both the BS and PL approach (SD ± 17.9 range; 50–100 mmHg).

### 3.2 Intraoperative visualization

**FIGURE 2:**
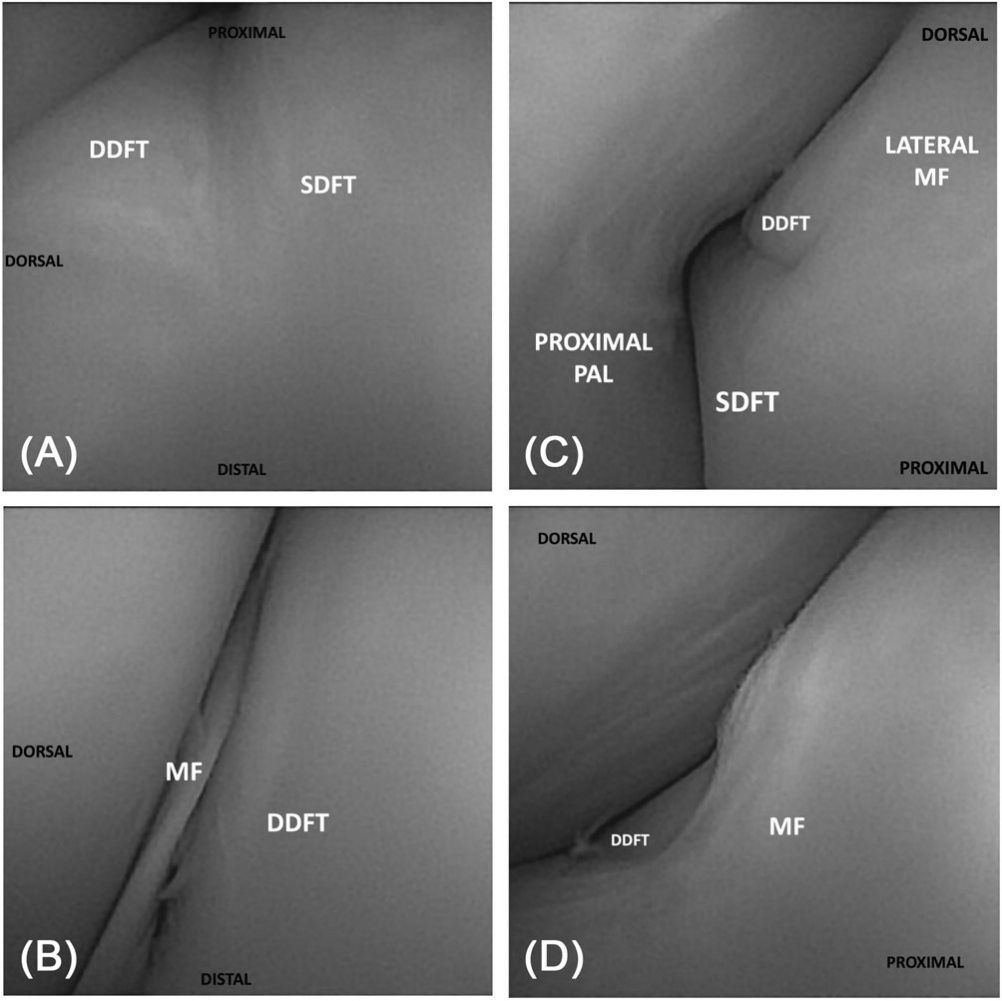
Needle tenoscopic visualization within the digital flexor tendon sheath (DFTS): (A) Lateral borders of the superficial digital flexor tendon (SDFT) and deep digital flexor tendon (DDFT) viewed proximally via the basisesamoid approach. (B) Dorsal border of the DDFT and distal border of the manica flexoria (MF) viewed proximally via the basisesamoid approach. (C) Lateral borders of the SDFT, DDFT, and palmar/plantar annular ligament (PAL) viewed distally via the proximolateral approach. (D) Dorsal aspect of the MF and DDFT viewed distally via the proximolateral approach.

Visualization sum scores for all intrathecal structures from both BS and PL approaches are presented in Table 1. The PL approach offered better visualization than the BS approach (*p* = .025). Visualization of the SDFT and DDFT from both a BS (*p* = .001) and a PL (*p* = .001) approach in the fetlock canal was better than visualization of the SDFT and DDFT in the proximal DFTS, where it was for the most part poor (Figure 2). The PL approach resulted in better visualization of the PAL than the BS approach (*p* = .001). Visualization of the PAL distal border was excellent from both BS and PL approaches. Visualization of the proximal border was excellent from the PL approach but it was never visualized from the BS approach.

The PL approach resulted in better visualization of the MF than the BS approach (*p* = .004) (Figure 2). Both approaches provided excellent visualization of the distal margin of the MF but the PL approach also provided excellent visualization of its medial and lateral margins, which were only partially visualized from the BS approach. Within the proximal DFTS, the lateral aspect of the SDFT was considered to be the body of the SDFT that was lateral to the MF, and the lateral margin of the manica flexoria was considered to be the junction between the SDFT and the MF.

From the sub‐MF approach, all borders of the DDFT had excellent visualization, both within the fetlock canal and within the proximal DFTS. The SDFT palmar border was never visualized from the sub‐MF approach. Within the fetlock canal, the SDFT dorsal, medial, and lateral borders had excellent visualization from the sub‐MF approach. Within the proximal DFTS, the SDFT dorsal and lateral border had excellent visualization from the sub‐MF approach. Visualization of the distal, medial, and lateral borders of the MF was also excellent; however, the proximal and distal borders of the PAL were never visualized. This approach was also the only approach to provide visualization of the mesotenons of the DDFT.

### 3.3 PAL desmotomy

**FIGURE 3:**
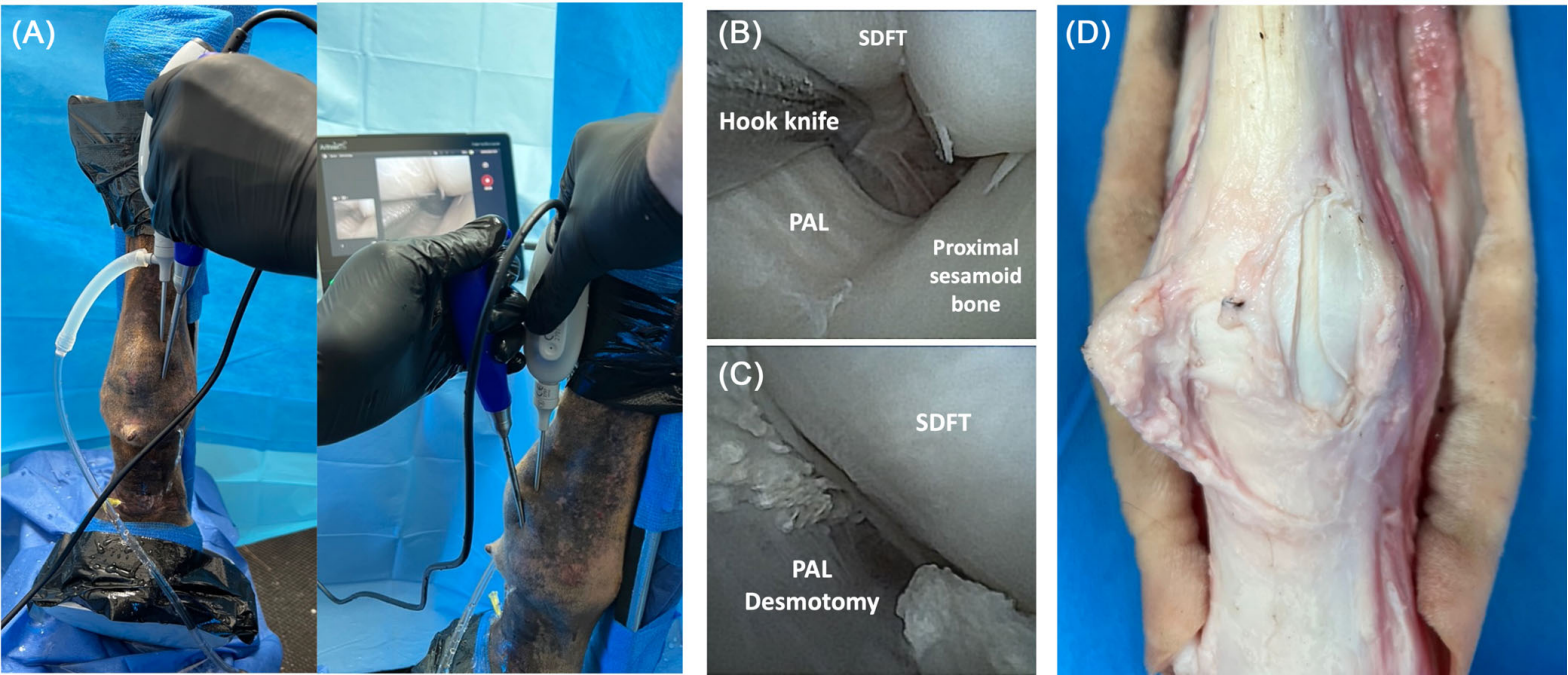
(A) External positioning of the hook knife for palmar/plantar annular ligament (PAL) desmotomy in a standing equine cadaver model under needle tenoscopic guidance. (B) The knife engaging the PAL. (C) The complete PAL desmotomy. (D) The PAL desmotomy seen on dissection.

The instrument portal to perform PAL desmotomy was created on the first attempt in 9/10 limbs; the skin incision had to be extended in one limb (Table 2). The mean time to create the instrument portal and transect the PAL was 6 min, 56 s (SD ± 2 min, 8 s; range 3 min, 13 s–9 min, 27 s). The PAL was successfully transected on the first attempt in 8/10 limbs and on the second attempt in 2/10 limbs, both of which required extending the desmotomy at its most distal aspect (Table 2). The PAL desmotomy was complete in all limbs (Figure 3D).

### 3.4 Iatrogenic damage, extravasation and dissection

**FIGURE 4:**
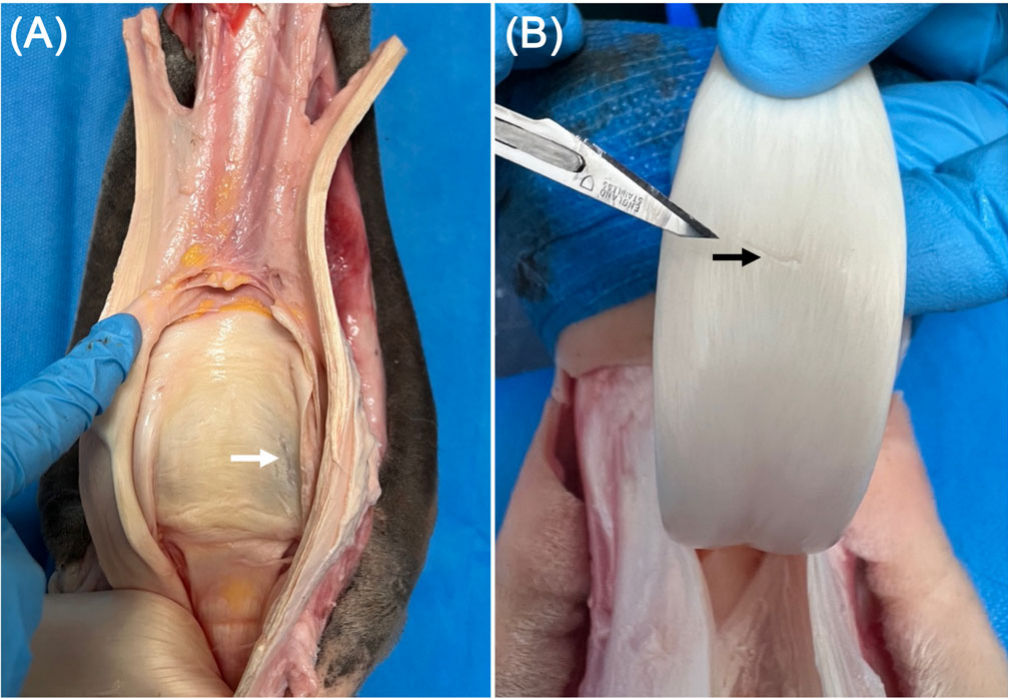
The iatrogenic damage most commonly observed during dissection following needle tenoscopy of the digital flexor tendon sheath. Excoriations of (A: white arrow) the proximal scutum, and (B: black arrow) the dorsal deep digital flexor tendon (DDFT).

Iatrogenic damage was recorded in four limbs following a BS approach and graded mild (superficial excoriation of proximal scutum in two limbs; superficial excoriation of the dorsal DDFT at the level of the fetlock canal in two limbs) (Figure 4). Iatrogenic damage was recorded in three limbs following a PL approach and also graded mild (superficial excoriation of proximal scutum in one limb; superficial excoriation of dorsal DDFT at the level of fetlock canal in two limbs) (Figure 4). Extravasation was present at three BS portals and one PL portal and, graded as mild on all occasions. Dissection of the DFTS found 19/20 portals to be in the correct location; one portal (BS) was located adjacent to the palmar digital neurovascular bundle; however, the palmar digital neurovascular bundle was intact. PAL thickness was 2 mm in 6/10 limbs and 3 mm in 4/10 limbs. No clinically significant iatrogenic damage was observed during needle tenoscopy or PAL desmotomy. Dissection confirmed that the DFTS was normal in all limbs.

## 4 DISCUSSION

This study describes two different approaches for needle tenoscopy in a standing equine cadaver model to visualize the intrathecal anatomy of the DFTS. It also describes a successful novel method of PAL desmotomy.

Traditional DFTS tenoscopy requires general anesthesia, which has a mortality rate of 0.9% for elective procedures.^29^ The concept of standing tenoscopy has been proven with other tendon sheaths^23^ and the results of this study indicate that needle tenoscopy could provide diagnostic visualization of clinically relevant anatomy and guide PAL desmotomy in the standing patient, thus avoiding the risks and costs associated with general anesthesia. Needle endoscopy of the DFTS has been described in a lateral recumbency model but this was not considered a viable alternative to traditional DFTS tenoscopy and PAL desmotomy.^25^ The poor image quality resulted in iatrogenic damage to the SDFT and incomplete PAL desmotomies.^25^ However, with this standing model using the larger diameter needle scope, the improved image quality and maneuverability facilitated better intraoperative visualization and complete PAL desmotomy in all limbs with no iatrogenic damage associated with the use of the hook knife.

All needle tenoscopy examinations were performed efficiently with a mean time of less than 9 min. A study assessing standing carpal tenoscopy found horses to tolerate splinting in a flexed position adequately for up to 10–15 min.^23^ The Kimzey leg‐saver splint used in this study aimed to provide stable partial fetlock flexion, to facilitate tenoscopy and PAL desmotomy. Splint modification and acclimatization may be necessary prior to surgery to assess tolerance on an individual horse basis.^23^

A further utility of the needle scope could be its use under general anesthesia to facilitate safe PAL transection in cases of severe PAL constriction where a traditional rigid arthroscope cannot be safely advanced through the constricted fetlock canal. The smaller diameter of the needle scope may allow positioning of the scope proximal to the PAL, allowing visualization of an instrument portal and passage of instruments intrathecally. The authors have successfully used the needle scope under general anesthesia in this way in severe clinical cases of PAL constriction, to guide PAL desmotomy; no additional measures were taken to perform the procedures in either case; however, ultrasonography could be considered to guide creation of the tenoscopic portals. The needle scope is a good alternative in clinical settings where smaller diameter arthroscopes (2.4 or 2.7 mm) are not readily available. Contrary to a study assessing DFTS tenoscopy using a smaller diameter needle scope,^25^ the larger diameter needle scope system provided an image quality sufficient to guide safe PAL desmotomy.

Overall visualization of the DDFT and SDFT in the fetlock canal was excellent but within the proximal DFTS visualization was poor. From a BS approach, the needle scope's short working length limited visualization of the most proximal extent of the DFTS. The large handpiece of the needle scope contacted the wide Kimzey hoof cup limiting maneuverability. The flexible nature of the needle scope made maneuvering between channels within the DFTS difficult or sometimes impossible, a previously reported complication of needle endoscopy.^23^, ^25^ Finally, the zero‐degree working angle removed the ability of the surgeon to periscope, as in traditional tenoscopy, to widen the field of view. From a PL approach, the proximal DDFT and dorsal surface of the SDFT could not be visualized as they were positioned under the MF, whereas the needle scope was positioned dorsal to the MF, thus limiting visualization of the DDFT and medial SDFT. If proximal DFTS pathology is suspected, needle tenoscopy of the DFTS would therefore provide only limited assessment.

The sub‐MF approach was not considered an important iatrogenic injury as tenoscopic instrument portals are created through the proximal MF reflection for debridement of lateral DDFT tears that extend proximally under the MF.^6^ The sub‐MF approach was the only approach to provide excellent visualization of the DDFT in the proximal DFTS and to visualize the mesotenons of the DDFT. The sub‐MF approach could be combined with the standard PL approach to increase the visualization of proximal intrathecal DFTS anatomy. Ultrasound guidance could be utilized when placing the cannula to facilitate safe placement of the needle scope in the sub‐MF position.

Longitudinal tears of the DDFT are a commonly diagnosed pathology of the DFTS^2^, ^4^, ^6^ and occur most commonly along the lateral border, originating in the fetlock canal.^2^, ^6^ Ultrasound and contrast radiography have limited sensitivity and specificity in diagnosing DDFT lesions.^2^, ^4^, ^9^, ^10^ Both needle tenoscopic approaches offered excellent visualization of the lateral border of the DDFT within the fetlock canal, highlighting a potential clinical application of needle tenoscopy as a diagnostic modality in cases where a diagnosis remains inconclusive following diagnostic imaging.

Manica flexoria tears commonly occur along the medial margin.^13^ Both approaches provided excellent visualization of the MF distal border; however, the PL approach allowed excellent visualization of the lateral and medial MF margins. From the BS approach, the Kimzey leg‐saver splint restricted needle scope maneuverability, the zero‐degree working camera angle and lack of rigidity limited visualization of the medial MF margin. The PL approach provided superior maneuverability and would be preferable in clinical situations where assessment of the MF is indicated. Surgical sterility is also easier to maintain from the PL approach, as the hand piece is elevated further from the ground surface. However, as ultrasonography and contrast radiography have high sensitivity and specificity for detecting MF lesions^9^, ^10^, ^13^ and considering the complexity of performing a MF resection we would not recommend needle tenoscopy in the standing patient where MF tears are suspected.

The BS approach failed to visualize the proximal PAL border due to the Kimzey leg‐saver splint obstructing the handpiece of the needle scope. For this reason, the PL portal was chosen to guide PAL desmotomy as it allowed excellent visualization of the proximal and distal borders of the PAL in all limbs. A PAL desmotomy is performed to treat PAL constriction and to facilitate instrumentation in constricted fetlock canals.^7^, ^8^, ^26^ Desmotomy of the PAL has been described under tenoscopic visualization,^26^ blind,^30^ under ultrasound guidance,^31^ and via a percutaneous thread‐transecting technique,^32^ with the latter two described as procedures that could be employed in the standing horse. Under needle tenoscopic guidance, complete desmotomy was achieved in all limbs without any associated iatrogenic damage. This is in contrast to ultrasound guided and percutaneous thread‐transecting techniques for PAL desmotomy, which resulted in incomplete desmotomy in 10% and 19% of limbs, respectively.^31^, ^32^ The advantage of needle scope‐guided PAL desmotomy is the ability to visualize the PAL transection and ensure a complete transection whilst avoiding iatrogenic damage. It may therefore be possible to perform needle scope‐guided PAL desmotomy in clinical cases.

No clinically significant iatrogenic damage occurred from either approach in this study. Superficial excoriation was identified on the scutum and DDFT. This was attributed to the use of the wide‐bore, high‐flow cannula used in this study to allow better distention of the DFTS and also due to its increased rigidity. This facilitated maneuverability but also likely increased the nonclinically significant iatrogenic damage, a similar finding for needle endoscopy.^23^ Some degree of needle scope flexibility could protect against iatrogenic damage especially if a horse were to move intraoperatively but this flexibility may limit the ability to move the needle scope between the tendons.

Limitations of this study include the small number of limbs and the fact that all DFTS were clinically normal and the majority of limbs were not from cob, draught, or pony breeds, in which PAL constriction is more common.^33^ The cadaveric limbs were also transected at the distal radius or tibia, potentially resulting in decreased tension of the flexor tendons, which may have increased visualization and needle scope maneuverability. The pastern region of the DFTS was not explored as lesions occur more commonly within the fetlock canal, or proximal DFTS.^2^, ^3^, ^4^ In the authors' opinion, if a lesion was suspected in the pastern region, then the case would not be suitable for standing needle tenoscopy due to the close proximity of the ground.

In conclusion, needle tenoscopy of the DFTS is a minimally invasive technique that allowed diagnostic visualization of selective intrathecal anatomy depending on the approach. Needle scope‐guided PAL desmotomy was successful in all limbs. Further work is required to determine the sensitivity and specificity with which lesions can be identified in clinical cases and the feasibility of performing the technique in standing live horses. Findings from this study provide the foundations for further investigation of the capabilities of nanoscopic endoscopy in equine surgery.

## AUTHOR CONTRIBUTIONS

Breen LJ, BVSc, MRCVS: Contributed to study design, study execution, data analysis and interpretation, and preparation of the manuscript. Stack JD, MVB, MSc, DVMS, DipECVS, FHEA, MRCVS: Contributed to study design, study execution, data analysis and interpretation, and preparation of the manuscript. Gillen AM, MA, MS, VetMB, CertAVP, DipECVS, FHEA, MRCVS, DACVS (Large Animal): Contributed to study design and preparation of the manuscript. Baldwin CM, BVetMed (Hons), CertAVP (ESST) (EOS), DipECVS, AFHEA, MRCVS: Contributed to study design, study execution, data analysis and interpretation, and preparation of the manuscript. All authors gave their final approval of the manuscript.

## FUNDING INFORMATION

None.

## CONFLICT OF INTEREST

The authors declare no conflicts of interest related to this report.
